# Investigation of a Truncated Aptamer for Ofloxacin Detection Using a Rapid FRET-Based Apta-Assay

**DOI:** 10.3390/antibiotics9120860

**Published:** 2020-12-03

**Authors:** Sondes Ben Aissa, Mohamed Mastouri, Gaëlle Catanante, Noureddine Raouafi, Jean Louis Marty

**Affiliations:** 1 Laboratoire BAE-LBBM, Université de Perpignan Via Domitia, 52 Avenue Paul Alduy, CEDEX 9, 66860 Perpignan, France; sondes.benaissa@fst.utm.tn (S.B.A.); gaelle.catanante@univ-perp.fr (G.C.); 2 Sensors and Biosensors Group, Laboratoire de Chimie Analytique et Electrochimie (LR99ES15), Faculté des Sciences de Tunis, Universitairé de Tunis El Manar, Tunis 2092, Tunisia; mohamed.mastouri@fst.utm.tn

**Keywords:** aptamer, ofloxacin, truncation, docking, FRET, hybridization, quenching, milk

## Abstract

In this work, we describe the use of a new truncated aptamer for the determination of ofloxacin (OFL), being a principal quinolone commonly used in both human and animal healthcare. Since the affinity of a 72-mer ssDNA sequence has been previously described without further investigations, this paper demonstrates the first computational prediction of the binding motif between this aptamer and OFL through in silico molecular docking studies. Besides, we suggest the application of the characterized recognition mechanism in a simple FRET (Förster Resonance Energy Transfer) pattern for the rapid aptasensing of the quinolone of interest. Accordingly, our approach harnesses the fluorescence quenching of the fluorescein-tagged aptamer (FAM-APT) induced by its partial hybridization to a tetramethyl rhodamine-labelled complementary ssDNA (TAMRA-cDNA). In such a structure, dye labels brought into close proximity act as a FRET pair. Upon ofloxacin addition, an affinity competition occurs to form a more stable FAM-APT/OFL complex, thus unquenching the FAM-APT signal. Interestingly, the recovered fluorescence intensity was found to correlate well with the antibiotic’s concentrations in the range of 0.2–200 μM in HEPES buffer, with a linear response that ranged between 0.2 and 20 μM. The rapid apta-assay achieved limits of detection and quantification of 0.12 and 0.40 μM, respectively. The truncated aptamer has also shown an improved specificity toward OFL than other quinolones, compared to the original full-length aptamer described in previous works. Finally, the practical application of the developed apta-assay was successfully confirmed to detect OFL quinolone in spiked milk samples, with satisfactory recoveries ranging between 97.4% and 111.4%.

**Highlights:**

1. A 72-mer truncated aptamer is investigated for the first time in the analysis of ofloxacin.
2. The optimal binding site responsible for OFL recognition is confirmed using molecular docking studies and FRET-based experiments.
3. A rapid, simple, and low-cost fluorometric apta-assay for the detection and quantification of ofloxacin is described.
4. Aptamer truncation enhanced the selectivity toward ofloxacin than other structurally comparable fluoroquinolones.

## 1. Introduction

Quinolones constitute a large class of synthetic antibiotic agents that are highly effective in the treatment of many types of infectious diseases [1]. Particularly, their fluorinated derivatives, referred to fluoroquinolones (FQs), are used worldwide, taking advantage of their broad medicinal activities against most Gram-negative bacteria and many Gram-positive bacteria [2]. The revolutionizing medicinal properties of FQs have increased their overuse, mainly in veterinary medicine for promoting the growth of animals and optimizing large-scale breeding programs. This excessive and inappropriate use is accelerating the emergence of antimicrobial resistance (AMR), which makes infections induced by microorganisms difficult to treat and sometimes incurable [3]. According to the World Health Organization (WHO), the AMR has become a global health concern since expected fatalities caused by multi-drug resistance will surpass 10 million by 2050 [4,5].

Among second-generation FQs, ofloxacin is primarily used in treating infections of the respiratory, digestive, and urinary systems in both humans and animals. Despite the remarkable features of this quinolone, its contribution to the expansion of bacterial resistance remains inevitable [6,7]. The consumption of animal-derived foodstuffs with high residual ofloxacin concentrations not only promotes the expansion of AMR but can also cause adverse reactions in the human body [8]. Therefore, it is crucial to control the level of fluoroquinolone residues in animal-derived foodstuffs before consumption. Many countries have set maximum residue limits (MRLs) of FQs in livestock products. For instance, Japan has established an MRL value of 10 ppb for ofloxacin [9]. While the European standards specify only limits for the sum of FQs between 300 and 800 ppb [10].

To ensure that the MRLs are not exceeded in various alimentary and environmental matrices, the development of sensitive diagnostic methods for on-site detection of FQ residues is highly needed. On the one hand, immunological and microbiological [11] screening assays provide rapid response regarding the presence of antibiotic residues, but they usually show low sensitivity and poor specificity. On the other hand, the legislative analytical techniques currently used for FQs analysis are the chromatographic methods, including high-performance liquid chromatography (HPLC), liquid chromatography-tandem mass spectroscopy (LC-MS/MS), and gas chromatography-mass spectrometry (GC-MS) [12]. Although highly sensitive and accurate, these techniques suffer from operational complexity, high cost, expensive instruments, long detection time, and require trained personals, which impairs their application for on-site analysis.

Therefore, noticeable progress has recently been made in the development of biosensing tools as potential alternatives to monitor antimicrobial drug residues in animal-derived food [13]. These biosensors are generally based on affinity assays and mainly make use of optical and electrochemical transducer systems [14]. To date, the most frequently described biosensors for antibiotic detection are those based on antibody/antigen affinity pairs [15]. However, there are fewer reports on detection assays designed specifically for quinolones in the recent literature [16].

Among affinity bioreceptors, aptamers have been long studied as a substitute of antibodies due to their numerous merits, including robustness, low cost, and reusability [17]. Their stable chemical structure allows the insertion of signaling probes and surface-binding groups in specific positions, which have been widely exploited for the development of aptamer-based biosensors and direct detection assays. Amidst a large choice of aptasensing strategies, optical aptasensors based on fluorescence approaches have recently evolved as an excellent candidate for rapid evaluation of antibiotics, owing to their superior sensitivity, selectivity, and ease of manipulation [18]. In fact, oligonucleotides can undergo conformational rearrangements upon adaptive binding with their targets, which provide a basis to induce fluorescent signaling/quenching with various mechanisms, such as energy transfer. For instance, Youn et al. [19] described a GO-based fluorescent aptasensor for the simultaneous detection of sulfadimethoxine, kanamycin, and ampicillin using three specific aptamers tagged with different fluorophores. Since the graphene oxide (GO) acts as a universal quencher, the desorption of oligonucleotides from GO sheets in the presence of antibiotics induced the fluorescence turn-on of each probe. In a similar FRET strategy, Dolati and colleagues [20] developed a fluorescent assay based on label-free aptamers and the fluorescence quenching ability of GO for the detection of enrofloxacin (ENR). The native fluorescence of this quinolone was quenched by adsorption on GO, whereas the ENR-aptamer complex showed less affinity toward GO. The method was very sensitive and able to detect ENR in milk with a LOD lower than the corresponding MRL.

Nonetheless, the drastic size difference between the small targets and aptamers is a main challenge to select, characterize, and validate aptamers through conventional SELEX procedures [21]. In-depth investigation on the interaction mechanism between aptamer and their targets is eagerly required, including the affinity, the interaction mode, as well as the structural changes of the aptamer before and after binding with analytes [22]. For instance, rational truncation of selected oligonucleotides can further help in identifying sequence fractions involved in the direct contact with the target, which reduces the possibility of cross-recognition. Alternatively, in silico methods can also be applied as useful complementary options for identifying or optimizing aptamers over small ligands in a cost- and time-effective way [23].

**Figure 1:**
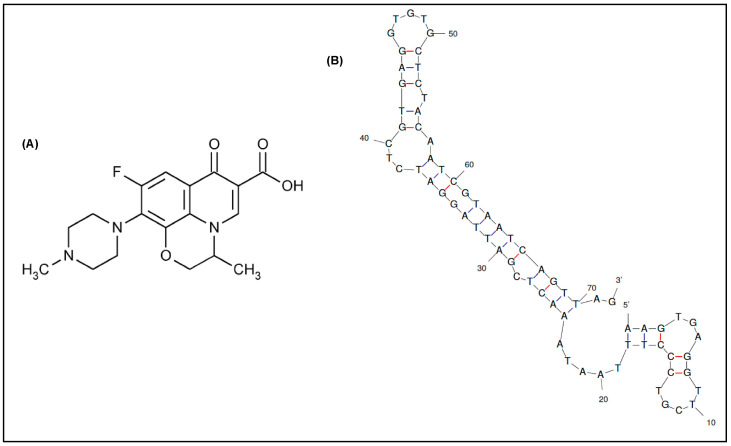
(**A**) Chemical structure of ofloxacin. (**B**) 2-D structure of the truncated DNA aptamer modeled by *Mfold* web server [27] (∆G = −24.36 kcal/mol).

In this context, the aptamers selected so far for ofloxacin recognition are limited, usually comprising long ssDNA sequences (up to 98 nucleotides). Three long aptamers selected by Reinemann and colleagues [24] through capture-SELEX have shown the strongest affinity toward ofloxacin, which is illustrated by dissociation constants in the low nanomolar range (K_D_ = 0.1–56.9 nM) [25]. However, due to the exceeded length of the aptamers obtained in vitro, difficulties could arise in its manipulation during the development of biosensing tools. While targeting small organic molecules, it is widely accepted that a way to enhance aptamers’ affinity and specificity is the truncation of non-essential nucleotides, thus improving the accessibility of targets to the aptamer, resulting in stronger aptamer-target complexes [26]. The same group had subsequently performed a number of truncation experiments to narrow down the optimal binding sites in each aptamer [25]. However, only the recognition region within one aptamer referred to as Q1 was confirmed, suggesting that the longer stem structure located at the 3′ side is responsible for binding to ofloxacin (Figure 1B). Accordingly, the same aptamer truncated to 72 nucleotides by sacrificing the 5′ primer region was able to keep approximately 60% of its initial affinity [25]. Though this result was reported without further investigations.

Based on previous findings, the current work intends first to study in silico the binding mechanism between the 72-mer truncated aptamer and the target quinolone, then to apply it in a rapid fluorometric apta-assay for ofloxacin screening in milk. The computational results were evaluated on the basis of affinity and selectivity using a FRET-based apta-assay. More particularly, we demonstrate the development of a structure switching aptamer assay for the quantitative detection of ofloxacin employing the quenching-unquenching mechanism. Hybridization of a fluorescein-labelled aptamer (FAM-APT) specific to OFL with a TAMRA-labelled complementary sequence (TAMRA-cDNA) brings the fluorophore and the quencher into close proximity, which results in maximum fluorescence quenching in optimized conditions. Upon the addition of the target antibiotic, the latter induces a conformational change of the aptamer through the formation of a FAM-APT/OFL complex, which partially recovers the fluorescence under optimized conditions. This proportional recovery of fluorescence with the concentration of target made it suitable to quantify OFL in both buffer and milk solutions.

## 2. Results

### 2.1. Molecular Docking Studies

**Figure 2:**
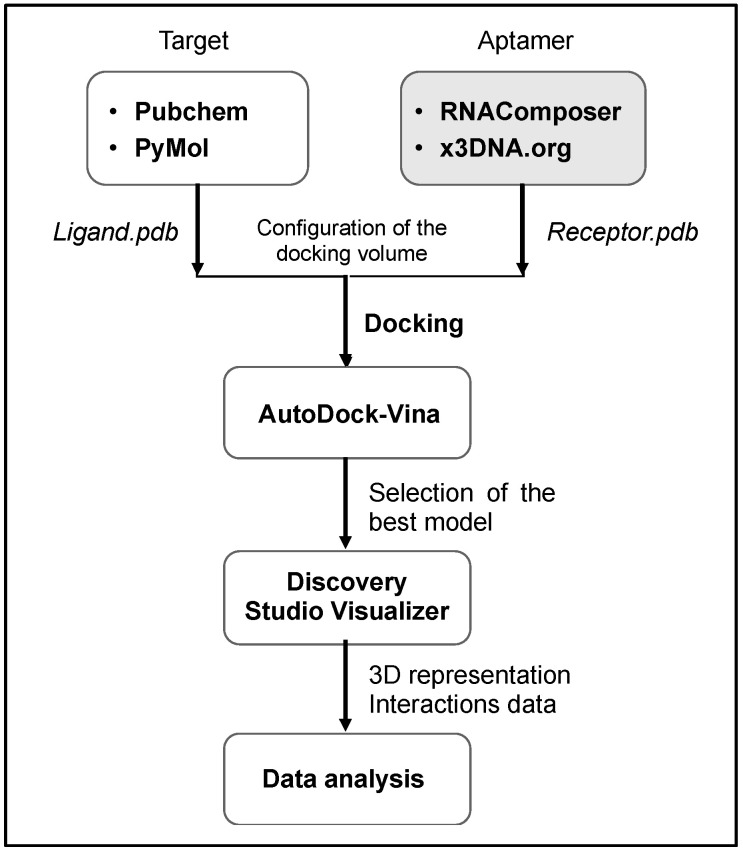
Molecular docking procedure as followed in the current work.

In silico study for molecular docking simulation was carried out according to a multi-stage procedure using a set of web-based and standalone programs as summarized in the following flowchart (Figure 2).

**Figure 3:**
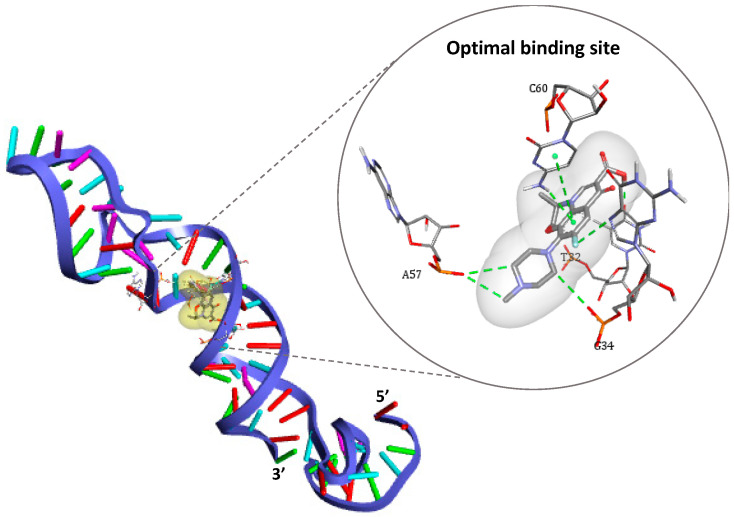
3-D structure of the DNA aptamer showing the optimal binding site for ofloxacin docking as represented by DS Visualizer.

After generation of the spatial coordinates of each 3-D structure in pdb format (DNA aptamer as receptor and ofloxacin as ligand), the main docking simulation was given by AutoDock Vina command line that outputs up to nine possible binding poses. In our case, all docking models have shown that nucleotide chains located between T27 and G34 from one side and C60-T65 from the other side are responsible for target capture. These models are classified per increasing Vina score (given in kcal.mol^−1^) that reflects the affinity strength, in addition to the corresponding values of root-mean-square deviations (RMSD) describing the changes in the conformation of the ligand in comparison to the model of best affinity (cf. Table S1). Subsequently, the binding site that has the lowest energy value of −7.9 kcal/mol^−1^ (−33.05 kJ/mol^−1^) and the best alignment (zero RMSD) was chosen herein as optimal for ofloxacin recognition (cf. Figure 3).

As shown in Figure 3, four nucleotides are involved in the recognition mechanism, two of which are placed in the 3′ primer region (T32 and G34), while others belong to the internally hybridized chain forming the longest stem structure (A57 and C60). These findings are in line with earlier truncation studies [25], which suggested that the same region containing the stem structure of the aptamer is responsible for OFL interactions.

Different types of interactions contribute to the binding of the antibiotic target, including hydrogen bonds, π-π stacking, halogen bounding (fluorine), and hydrophobic interactions. Furthermore, the simulation demonstrated that Watson–Cricks bonds in the DNA helix stabilizes the formed complex via Van Der Walls interactions (see details in Figure S1).

In light of this recognition mechanism, we presume that the competition between ofloxacin and a complementary ssDNA to the peripheral 3′ region should be applicable to detect the target via FRET signaling. In this strategy, aptamer/target binding unwinds the base pairing of the aptamer/cDNA and favors the aptamer intra-hybridization for OFL capture.

### 2.2. FRET-Based Apta-Assay

**Figure 4:**
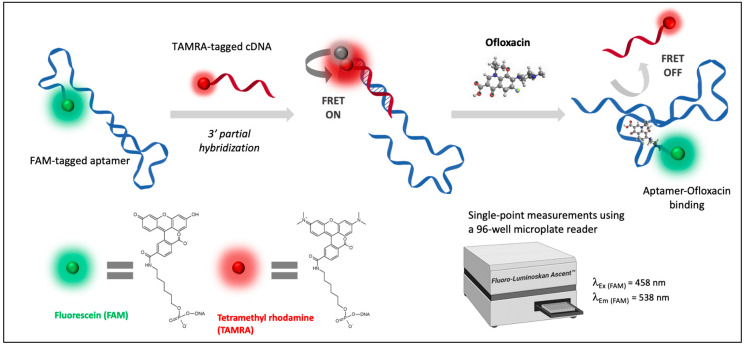
Schematic principle of the FRET-based apta-assay for the detection of ofloxacin.

To experimentally validate the docking results, the FRET-based apta-assay was conducted as illustrated in Figure 4. For rapid assessment, fluorescence response was recorded by a microplate reader through single-point measurements that correspond to fluorescein emission.

Preliminary optimizations were performed to choose the most convenient length of complementary DNA. This quencher should cover at least a part of the optimal recognition site to achieve the fluorescence recovery via TAMRA-cDNA release. Accordingly, ofloxacin complexation should exhibit a favorable binding energy that prevails over the duplex energy. A set of complementary sequences with different lengths was preliminarily tested, including 30-, 15-, and 12-mer cDNAs. Upon target addition after quenching, no fluorescence recovery was obtained with the 30-mer cDNA-TAMRA, while negligible recoveries were recorded using the 15-mer sequence. Only the 12-mer complementary sequence revealed a promising response following ofloxacin addition. Consequently, this shortest probe was retained to develop the apta-assay.

#### 2.2.1. UV Spectroscopy Characterization

Typically, the UV absorption spectrum of oligonucleotides shows a broad band in the UV region (200–300 nm) with maximum absorption at 260 nm. This maximum is a consequence of the chromophores in purine (adenine and guanine) and pyrimidine (cytosine and thymine) moieties responsible for the electronic transitions [28]. Hence, UV measurements in the range of 220–310 nm were performed, aiming to qualitatively characterize the structure switching of aptamer duplex into aptamer-OFL complex.

The UV absorption of FAM-APT (0.25 µM) was measured in the absence and presence of complementary DNA (0.5 µM) in the same conditions of the assay, and after target addition at a relatively high concentration (100 µM) to the DNA duplex. A fixed sample volume of 3 µL was used for each solution after stepwise additions to measure the corresponding UV absorption using the nanodrop cuvette.

**Figure 5:**
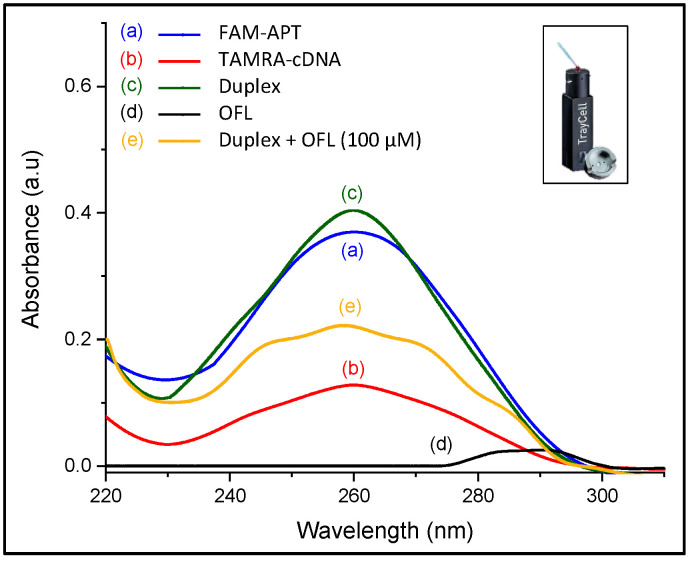
UV characterization of conformation changes in ssDNA sequences (inset: Photo of the Nanodrop cuvette used for the measurements).

As for ofloxacin, its aromatic structure emits a characteristic absorption signal at 290 nm. As shown in Figure 5, the 72-mer aptamer gives a higher maximum absorption intensity at 260 nm than the 12-mer complementary DNA due to their difference in sequence length. Upon hybridization, this peak has slightly increased since the number of chromophoric nucleotides arises in the duplex format. However, the addition of ofloxacin alters the partial dsDNA structure and displaces the electronic transitions to show multiple broad bands relative to the presence of the three molecular entities in the solution, which have not been investigated. The change in the UV absorption characteristics of the DNA duplex strongly reveals the target-induced complex formation [29], which is a preliminary indication that the recovery response was due to the docking between the target and aptamer.

#### 2.2.2. Apta-Assay Optimization

Experimental conditions substantially affect the analytical performance of the developed assay; therefore, all parameters were carefully investigated and optimized. Pertinent parameters, on which the FRET mechanism strongly depends, are related to the acceptor (FAM) and donor (TAMRA) quantities, as well as the interaction time between all molecular partners. The fluorescence intensity of the FAM probe was taken as an indicator throughout the apta-assay optimization.

##### Aptamer Fluorescence

The working concentration of FAM-APT in each well was fixed at 0.25 μM. This final concentration corresponds to a quantifiable fluorescence signal of 20.7 ± 2.1 a.u. (see Figure S2). Lower concentrations of aptamer would give a smaller detection range with low fluorescence signal after the quenching step, which is hardly distinguishable from buffer interference in diluted working solutions. On the other hand, higher aptamer concentrations may increase the output signal but ultimately result in lower sensitivity and higher cost of the assay. Besides, the selected final concentration of 0.25 µM was the best compromise to afford the 72-mer aptamer quantity and avoid using many batches that would alter the assay reproducibility.

**Figure 6:**
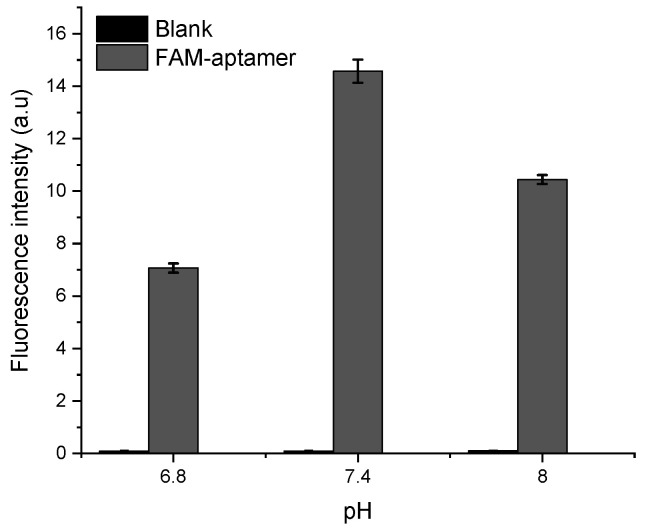
pH optimization based on the FAM-APT fluorescence intensity at 0.25 µM.

In a second step, the FAM probe signal was tested in HBB with different pH of 6.8, 7.4, and 8 to assess the optimal acidity for the apta-assay. The results shown in Figure 6 demonstrate that the fluorescence intensity is the highest at pH 7.4. This can be explained by the intrinsic pH-dependent charges of fluorescein, since it can be present in solution under different prototropic forms [30].

##### Quenching Step

The quenching step relies on the efficiency of energy transfer between the dye probes. It is crucial to increase the quenching efficiency to observe a sensitive fluorescence turn-on after target addition.

- *Molar ratio FAM/TAMRA*

**Figure 7:**
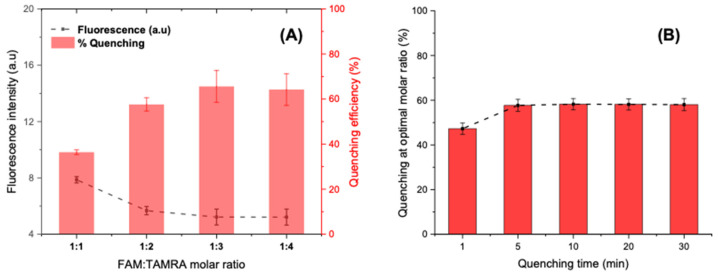
(**A**) Optimization of the FAM-APT-to-TAMRA-cDNA molar ratio for a maximum quenching at a fixed hybridization time of 5 min. (**B**) Optimization of the quenching time at an optimal molar ratio of 1:3.

Various concentrations of complementary DNA sequences were optimized against the fixed concentration of the FAM-labelled aptamer (25 pmol/well). Accordingly, the molar ratios of FAM-aptamer: TAMRA-cDNA sequences were set at 1:1, 1:2, 1:3, and 1:4. The fluorescence intensities were recorded on microplate reader after five minutes of interaction. The optimization results illustrated in Figure 7A show a steady increase in the quenching efficiency (%) when adding excess of TAMRA-cDNA until the 1:3 ratio, where more than 60% of the initial fluorescence is quenched. However, upraising the molar ratio to 1:4 does not show significant improvement. Hence, the optimized ratio with low background signal was selected to be 1:3 for further experimentation.

- *Quenching time*

At a fixed molar ratio, the fluorescence intensity was controlled during 30 min after the introduction of the quencher (75 pmol). The quenching results shown in Figure 7B suggest that the FRET occurs instantly as soon as the two fluorophores meet in solution by a contact quenching. Afterwards, the hybridization rapidly improves the quenching mechanism, which becomes more intense and stable after only 5 min and reaches a percentage of 60% that remains constant even after 30 min. Subsequently, 5 min was chosen as an optimal time for FRET activation via partial hybridization.

##### Ofloxacin Incubation

**Figure 8:**
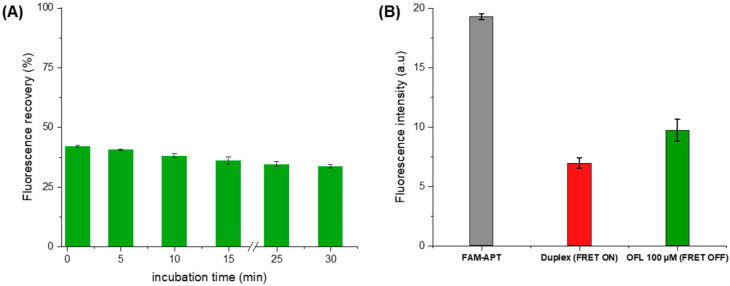
(**A**) Optimization of the ofloxacin incubation time as a function of fluorescence recovery (**B**) Fluorescence intensity at each aptasensing step in optimized conditions (5 min of OFL incubation).

Under the above optimal conditions, the fluorescence response was recorded for an OFL concentration of 100 μM (Figure 8A). Control measurements were carried out with each assay to calculate the percentage of fluorescence recovery. The *FL recovery* was calculated based on FAM’s fluorescence measurements after quenching as the following equation:

(1) $$\%\;FL\;recovery=\frac{F_{2}-F_{1}}{F_{1}}\times 100,$$

- *F*_1_: *FL* intensity after quenching;
- *F*_2_: *FL* intensity after ofloxacin addition.

Upon the addition of OFL (100 µM), we observe a partial recovery of the FAM fluorescence indicating the inhibition of energy transfer between the fluorescent pair (Figure 8B). This de-quenching refers to the preferential formation of a stable FAM-APT/OFL complex, validating the detection concept. Subsequently, the structure switching signaling apta-assay was extended to the detection of a larger range of ofloxacin concentrations.

### 2.3. Ofloxacin Aptasensing

**Figure 9:**
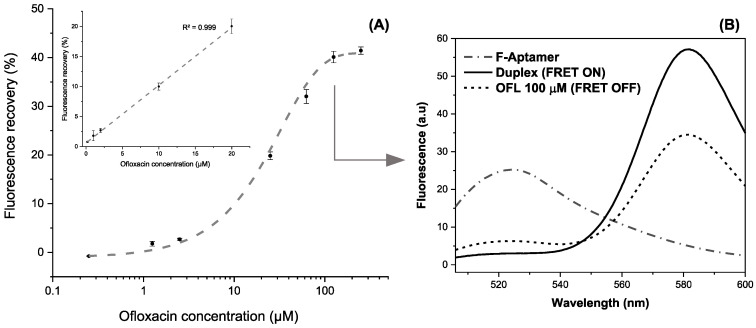
(**A**) Calibration curve in the range of 0.2–200 μM of OFL in the logarithmic scale (inset: Linear working range between 0.2 and 20 µM) (error bars are obtained with *n* = 3) (**B**) Fluorescence spectra snapshotting the different apta-assay steps for a fixed target concentration.

The incubation of increasing target concentrations between 0.2 and 200 µM prepared in HBB reveals a logarithmic variation of the recovered fluorescence (R^2^ > 0.998). The maximum recovery rate was obtained starting from 100 µM, where a plateau is reached at ~42% of fluorescence recovery (Figure 9A). The linear dynamic range extracted from these results varies between 0.2 and 20 µM following the equation Y = 0.96X + 0.59 (R^2^ > 0.999). The aptasensing platform shows high reproducibility illustrated by a relative standard deviation (RSD) of 0.5% based on three independent measurements in different micro-wells.

Therefore, the analytical performances of the detection system were concluded from the linear working curve. The plot’s slope (s) was used to calculate a sensitivity of 0.59 ± 0.02% · µM^−1^. Limits of detection (LOD) and quantification (LOQ) were obtained based on 3 and 10σ/s, respectively. The standard deviation (σ) of the instrument was estimated by six repetitions of blank samples containing FAM-APT (100 µL), TAMRA-cDNA (50 µL), and HBB (50 µL). Hence, the LOD and LOQ are achieved as 0.12 and 0.40 μM, respectively.

To highlight the quenching-unquenching mechanism occurring in a single well, Figure 9B shows an example of the fluorescence emission spectra after each interaction step, recorded between 500 and 600 nm. The FAM-labelled aptamer displayed a maximum emission signal at 525 nm that disappears upon duplex formation, while the FRET enhances the fluorescence of TAMRA at 580 nm, being the energy acceptor. Successively, the addition of ofloxacin at 100 µM triggers the aptamer folding and partially dissociates the complementary probe to recover a proportion of the FAM fluorescence. It is worth mentioning that slight changes of fluorescence intensities and corresponding wavelengths between single-point and continuous measurements are imputable to the instrumental difference in spectrofluorometers’ capabilities.

### 2.4. Selectivity of the Apta-Assay

**Figure 10:**
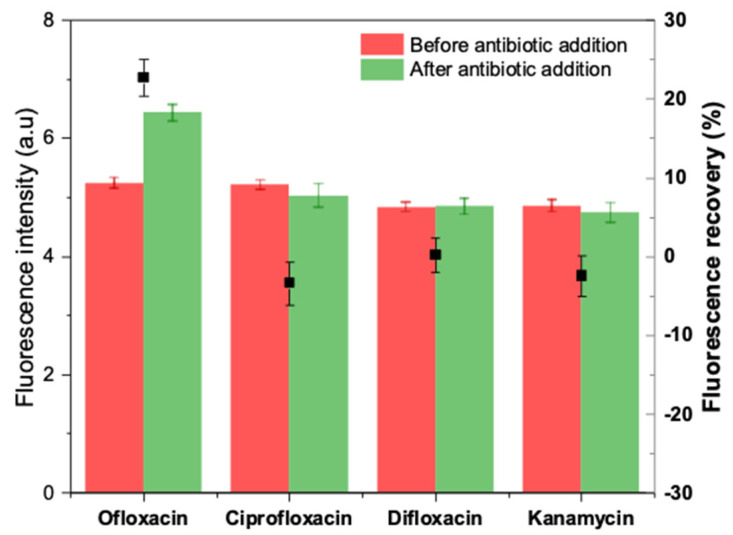
Selectivity of the apta-assay in the presence of other interfering antibiotics.

Potential interferents, including other fluoroquinolones (ciprofloxacin and difloxacin) and an aminoglycoside antibiotic (kanamycin), were tested in the same experimental conditions to evaluate the selectivity of the developed apta-assay. Each antibiotic was dissolved in HBB (pH 7.4) at a final concentration of 20 µM and incubated individually with prepared duplex. The obtained results are summarized in Figure 10.

After 5 min of incubation, negative fluorescence recoveries were obtained in the case of kanamycin and ciprofloxacin, while a blank-like response was recorded with difloxacin. On the other hand, ofloxacin caused a visible unquenching by turning on the fluorescence by 20% approximatively. Hence, the selectivity test demonstrates that the truncated aptamer-based method can distinguish OFL from either structurally comparable quinolones or other antibiotic classes.

### 2.5. Milk Samples Analysis

The practical application of the proposed apta-assay was investigated by determining ofloxacin in spiked milk samples. As shown in Table 1, recovery from 97.4% to 111.4% was obtained in the determination. Moreover, the relative standard deviation (RSD) was as low as 4%, demonstrating that the FRET aptasensing could meet the requirement for practical analysis in food matrices.

## 3. Discussion

The results of molecular docking confirmed that the recognition mechanism does not involve the totality of the aptamer sequence. Therefore, irrelevant regions can be discarded from the original sequence in order to enhance the specificity of the aptamer and subsequently push down the cost of this biomimetic bioreceptor. Though, the aptamer folding in the direct vicinity of the optimal binding site should be maintained after truncation since the Watson–Crick bonds have been shown to enhance the stability of the target-induced complex. As a first application, we describe herein the use of the 72-mer aptamer in a fluorometric assay to validate experimentally the molecular docking findings. Further investigations could be conducted with a 49-mer sequence sacrificing the A1-A23 nucleotides at 5′ region. This reasonable truncation would not alter the binding site structure.

The proposed aptameric assay consists of a two-step mechanism that involves an affinity competition between a short complementary single-stranded DNA and the target antibiotic within a 96-well microplate. Briefly, the FAM-tagged aptamer interacts first with the TAMRA-labelled cDNA of 12 nucleotides. The duplex structure is more stable than free cDNA sequences. The subsequent hybridization allows energy transfer via FRET since both fluorescent labels are found to be in a favorable orientation and proximity. Therefore, TAMRA-cDNA acts as a fluorescence quencher. In a second step, ofloxacin was added to the reaction medium, initiating an affinity competition with paired bases in the specific binding site of aptamer. Upon ofloxacin recognition, the aptamer-OFL complex disrupts the DNA duplex and recovers FAM fluorescence through TAMRA release. Hence, the recovered fluorescence intensity was demonstrated to be proportional to the final concentration of ofloxacin. This correlation was assessed by rapid single-point fluorescence measurements under optimized conditions.

One might notice that aptamer complexation to ofloxacin could not be thermodynamically more stable than a 12-mer dsDNA conformation since this small target engages only four principal bonds in optimal docking. However, this equilibrium displacement can be explained by the strong contribution of the aptamer’s internal base pairing. This mechanism suggests that aptamer folding into its intrinsic 3-D conformation is favored in the presence of OFL rather than its external hybridization to cDNA. In other words, the synergy between internal hybridization that engages more than 12 base pairs (see Figure 1) and ofloxacin affinity is responsible for the duplex breakdown.

Overall, the developed FRET-based apta-assay showed acceptable analytical response and demonstrated a high selectivity towards ofloxacin. However, relatively high limits of detection were obtained probably due to the limitations of the fluorescent transducers in this detection scheme.

Unlike previous reports that employ the full-length aptamer (97-mer), this method did not show cross-activity to other comparable fluoroquinolones (ciprofloxacin and difloxacin). Hence, the 72-mer sequence may be advantageous for a specific detection if only OFL is targeted.

It is worth mentioning that the developed apta-assay constitutes experimental evidence of the optimal recognition site, firstly determined by computational simulation. The current study’s primary objective was to investigate the interaction mode of a shorter aptamer with the target quinolone. Although the achieved analytical performances are not as competitive as the previous quantification methods (Table 2), the assay results provide new insights into ofloxacin aptasensing. Such findings open the path to more sensitive detection patterns using the same or shorter aptamers in other detection strategies, including optical and electrochemical approaches.

Nonetheless, the assay’s application in pretreated and spiked milk samples showed good recoveries and high reproducibility (RSD lower than 4%). More importantly, real sample analysis proved the applicability of the developed apta-assay in a relatively complex food matrix, representing an additional asset to the high specificity of truncated aptamers toward ofloxacin in a different medium than the buffer.

The comparison of the analytical properties of various methods for OFL analysis is summarized in Table 2. Only the electrochemical aptasensor [31] shows a faster response compared to our apta-assay. This is mainly due to different signaling strategies (electron transfer vs. photon transfer). Whereas, the present fluorometric assay requires the lowest time (5 min) to detect ofloxacin compared to other optical aptasensing platforms. Therefore, it can be useful as a rapid and affordable screening method to judge the presence or not of this fluoroquinolone at a concentration exceeding the LOD of 4.33 ppm (0.12 µM). Furthermore, the FRET assay owes the advantage of giving a rapid response without any required time for platform preparation. It avoids intricate and time-consuming fabrication processes of advanced electrochemical platforms.

In addition, to the best of our knowledge, this work reports the first proof of concept of using the 72-mer truncated aptamer to detect ofloxacin, supported by a molecular docking simulation.

## 4. Conclusions

To summarize, this work reports a rapid and cost-amenable aptasensing approach for the determination of ofloxacin in buffer and milk samples based on the specificity of a new 72-mer truncated aptamer. The 3-D structure, optimal binding site, and types of interactions of this oligonucleotide with OFL were successfully predicted by a comprehensive in silico study and found to be in line with experimental findings. Subsequently, this new insight into the molecular docking of anti-OFL aptamers is able to open new paths for further controlled truncations. Future work should focus on application of the minimal fragments including not only reactive nucleotides but also the stabilizing backbone chain.

The developed method detects ofloxacin in the ppm concentration range, which is not in compliance with MRLs for industrial applications. Alternatively, one way to improve the sensitivity of the described apta-assay is to use 2-D nanomaterials as universal quenchers, such as graphene oxide. This approach is currently being investigated to improve the assay sensitivity and reach competitive detection limits.

Finally, the reported findings would encourage research on further ofloxacin aptasensing studies that are currently slowed down because of the short aptamers’ paucity.

## 5. Materials and Methods

### 5.1. Materials and Reagents

All the chemicals for buffer preparation; HEPES sodium salt (≥99.5%), magnesium dichloride (MgCl_2_), potassium chloride (KCl), hydrochloric acid (HCl), sodium hydroxide (NaOH), and methanol (99.8%) were purchased from Sigma-Aldrich (Saint-Quentin-Fallavier, France) and used without further purification.

Ofloxacin (≥99% HPLC) was also obtained from Sigma-Aldrich and a 1 mg · mL^−1^ stock solution was prepared in deionized water, which was subsequently diluted in HEPES binding buffer (HBB) at an optimal pH of 7.4 (50 mM HEPES, 2 mM MgCl_2_, 120 mM NaCl). As for other antibiotics, kanamycin A, ciprofloxacin hydrochloride, and enrofloxacin were procured from VWR (Fontenay-sous-Bois, France).

The 72-mer aptamer labelled with fluorescein (3′-FAM) and its 12-mer complementary sequence modified in antiparallel position with tetramethyl rhodamine (5′-TAMRA) were synthetized by Microsynth (Balgach, Switzerland). Corresponding nucleic acid sequences are the following:

Aptamer: 5′AAG TGA GGT TCG TCC CTT TAA TAA ACT CGA TTA GGA TCT CGT GAG GTG TGC TCT ACA ATC GTA ATC AGT TAG -FAM 3′

cDNA: 5′ TAMRA- CTA ACT GAT TAC 3′

The milk sample was purchased from a local market in Perpignan, France. The preliminary preparation of milk solutions was performed by a three-step pretreatment; thermal deactivation of proteins, lipid depletion with methanol, and a final centrifugation as described in our previous paper [37].

### 5.2. Apparatuses

All fluorescence measurements were performed in the standard 96-well opaque microplates (Thermo Fisher Scientific, Denmark). Fluoroskan Ascent FL 2.6 equipped with an Ascent software version 2.6 (Thermo Scientific, Finland) was used to carry out the FAM’s fluorescence single-point measurements with excitation and emission wavelengths of 485 and 538 nm, respectively. The final volume per well was fixed at 200 μL.

Before each experiment, the aptamer solution was placed in a thermocycler (Mastercycler personal Eppendorf VWR, Leuven, Belgium) with the following temperature profile: heating at 90 °C for 8 min to the initial denaturation step, followed by a structure maintain step at 4 °C for 5 min, then a stabilization step at room temperature for 15 min.

A UV-visible spectrophotometer (UV-1800, Shimadzu, Japan) equipped with the TCC controller (TCC 240A) was used to measure the absorption characteristics of FAM-APT, DNA duplex, and target-induced aptamer folding. For this purpose, a Nanodrop cuvette (Traycell, Hellma) was used to record UV spectra with minimal samples volume fixed at 3 µL and a dilution factor cap of 10.

Fluorescence spectroscopy measurements for concept validation were carried out using an FP-8300 Spectrofluorometer from JASCO International Co.(Tokyo, Japan).

### 5.3. Molecular Docking

The molecular docking was performed according to the process illustrated in Figure 2. The initial atomic coordinates of ofloxacin were modeled based on its 3-D structure available in PubChem database, then validated in PyMol (V2.4.0, Schrödinger LLC). Regarding aptamer preparation, the RNAComposer web-sever [38] was first used to obtain the 3-D structure of the derived RNA (where thymine bases (T) are replaced by uracil (U)). Afterward, the platform x3DNA.org [39] allowed the simulation of the 3-D DNA aptamer in pdb (protein data base) format.

Subsequently, the AutoDock vina software (V 4.0) enabled the docking simulation between ofloxacin and the recognition region of aptamer. For docking, polar hydrogen atoms were added to the aptamer binding region and its nonpolar hydrogen atoms were merged using AutoDock Tools. Finally, the program Discovery Studio Visualizer (BIOVIA) [40] was useful to generate high-resolution docking images displaying binding interactions.

### 5.4. Fluorescence Measurements

All solutions were prepared in HEPES binding buffer (HBB, 50 mM), which is obtained by dissolving 50 mM HEPES sodium salt, 2 mM MgCl_2_, and 120 mM NaCl. The pH of HEPES buffer was adjusted to 7.4 using few drops of concentrated NaOH and HCl solutions (1 M). For fluorescence measurements, a final volume of 200 μL was kept fixed in the microwells throughout the optimization and analysis experiments (100 µL of FAM-APT, 50 µL of quencher, and 50 µL of target taking into account dilution factors while preparing standard solutions). The fluorescent measurements were performed using Fluoroskan Ascent FL 2.6.

A preliminary optimization of the aptamer quantity per well was first conducted. Therefore, fluorescence intensities of FAM-APT standard solutions were recorded between 0.05 and 0.8 μM to select an optimal concentration of practical use.

Afterward, quenching experiments under different conditions were carried out to optimize two critical parameters; (i) Aptamer: quencher ratio and (ii) quenching time. Accordingly, the ratios of FAM-aptamer: TAMRA-cDNA were set at 1:1, 1:2, 1:3, and 1:4. The optimized ratio with low background signal was selected for further experimentation. As well, fluorescence intensities were recorded every 5 min until stabilization to assess the best quenching time. To calculate the quenching percentage, control experiments were performed without the addition of quencher while keeping all other conditions constant.

After quenching, the DNA duplex formed at the optimized ratio was allowed to incubate with different ofloxacin concentrations (0.2 to 200 μM) for an optimized time interval, then the recovered fluorescence upon FRET pair dissociation was recorded. The solution in microwells was mixed by pipetting and kept reacting up to 5 min until an observable fluorescence recovery of FAM was obtained. Similarly, six control microwells measured without OFL addition served as a blank for FL recovery calculations.

### 5.5. Milk Samples Preparation

Milk samples were prepared using three main steps. First, we spiked three milk volumes of 900 μL with 100 µL of OFL standard solutions to a final concentration of 10, 50, and 125 µM. Spiked samples were heated in a water-bath at +40 °C for 30 min as the preliminary pretreatment step. Next, each milk sample was mixed with HPLC-grade methanol (1/3 *v*/*v*) for lipid and protein depletion. A total volume of 1.5 mL was then centrifuged for 5 min at 6000× *g* rpm at RT. After centrifugation, both the fatty cream and heavy precipitate were discarded, to recover around 800 µL of the supernatant. The pH of each supernatant was adjusted to 7.4 before direct analysis using the developed apta-assay.

## Figures and Tables

**Table 1:** Analysis of spiked milk samples using the developed FRET apta-assay.

Sample	Added OFL (mM)	Found OFL (mM)	RSD (%) (*n* = 3)	Recovery ^1^ (%)
1	10	10.6	3.9	105.5
2	50	55.7	4.1	111.4
3	125	121.7	4.0	97.4

^1^ (Found OL/Added OFL) × 100.

**Table 2:** Comparison with earlier optical aptasensing platforms for OFL determination.

Detection Strategy	Aptamer Length (nt)	Linear Range (M)	LOD (M)	Detection Time (min)	Real Sample	Ref.
Electrochemical aptasensor based on modified glassy carbon electrode with Au NPs/Aptamer	98	5 × 10^−8^–2 × 10^−5^	1 × 10^−9^	0.4	Tap water and effluent of sewage plant samples	[31]
Colorimetric apta-assay using label-free aptamers and the aggregation of gold nanoparticles	98	2 × 10^−8^–4 × 10^−7^	3.4 × 10^−9^	70	Tap water and Artificial urine	[32]
Photoelectrochemical aptasensing based on the use of a nanocomposites (TiO_2_ NTs, polydopamine and Ag_2_S NPs)	98	5 × 10^−12^–1 × 10^−7^	0.75 × 10^−12^	60	Milk	[33]
Fluorescent aptasensor based on the aggregation of AuNPs and its effect on quenching the fluorescence of Rhodamine B	98	2 × 10^−8^–3 × 10^−7^	1.66 × 10^−9^	50	Milk	[34]
Luminescence-based aptasensor using rolling circle amplification and magnetic separation	98	5 × 10^−11^–5 × 10^−8^	3.21 × 10^−11^	120	Chicken and pork	[35]
Fluorometric assay by using an aptamer and SYBR Green I	98	1.1 × 10^−9^–200 × 10^−9^	0.34 × 10^−9^	<40	Tap water, river water and artificial urine	[36]
FRET-based competitive apta-assay using FAM-labelled truncated aptamer	72	2 × 10^−7^–2 × 10^−5^	1.2 × 10^−7^	5	Milk	This work

## Supplementary Materials

The following are available online at https://www.mdpi.com/2079-6382/9/12/860/s1, Figure S1: Types of interactions between ofloxacin and aptamer upon recognition (model 1), as shown by DS visualizer (First letter indicates the nucleotide type, A is referred to Aptamer, and the number states the nucleotide’s position in the aptamer sequence); Figure S2: Optimization of initial FAM-Aptamer concentration, Table S1: Results of the molecular docking using AutoDock Vina program.
