# Supplementary material for: Investigation of a Truncated Aptamer for Ofloxacin Detection Using a Rapid FRET-Based Apta-Assay
Source: Antibiotics (Basel). 2020 Dec 3;9(12):860. doi: 10.3390/antibiotics9120860 (PMC7761777; doi:10.3390/antibiotics9120860)
Supplement: Supplementary file 1 [file antibiotics-09-00860-s001.pdf]

antibiotics

Investigation of a Truncated Aptamer for Ofloxacin Detection Using a Rapid FRET-Based Apta-Assay

Sondes Ben Aissa [1,2], Mohamed Mastouri [2], Gaëlle Catanante [1], Noureddine Raouafi [2,*] and Jean Louis Marty [1,*]

1 Laboratoire BAE-LBBM, Université de Perpignan Via Domitia, 52 Avenue Paul Alduy, CEDEX 9, 66860 Perpignan, France; sondes.benaissa@fst.utm.tn (S.B.A.); gaelle.catanante@univ-perp.fr (G.C.)
2 Université de Tunis El Manar, Faculté des Sciences de Tunis, Laboratoire de Chimie Analytique et Electrochimie (LR99ES15), Sensors and Biosensors Group, Campus Universitaire de Tunis El Manar, 2092 Tunis, Tunisia; mohamed.mastouri@fst.utm.tn
* Correspondence: noureddine.raouafi@fst.utm.tn (N.R.); jlmarty@univ-perp.fr (J.-L.M.); +33-4-68-66-22-54 (J.-L.M.); +216-55-985-599 (N.R.)

1. Molecular Docking study

Table S1. Results of the molecular docking using AutoDock Vina program.

Model	Affinity (kcal/mol)	Distance from best model	
		RMSD l.b.	RMSD u.b.
**1**	-7.9	0.000	0.000
**2**	-7.7	2.696	7.273
**3**	-7.7	2.877	3.662
**4**	-7.6	3.440	7.503
**5**	-7.6	9.197	11.298
**6**	-7.6	8.928	11.866
**7**	-7.5	11.057	14.070
**8**	-7.5	9.293	12.021
**9**	-7.3	9.970	10.970

RMSD: Root-Mean-Square Deviation. l.b.: lower bond; u.b.: Upper bond.

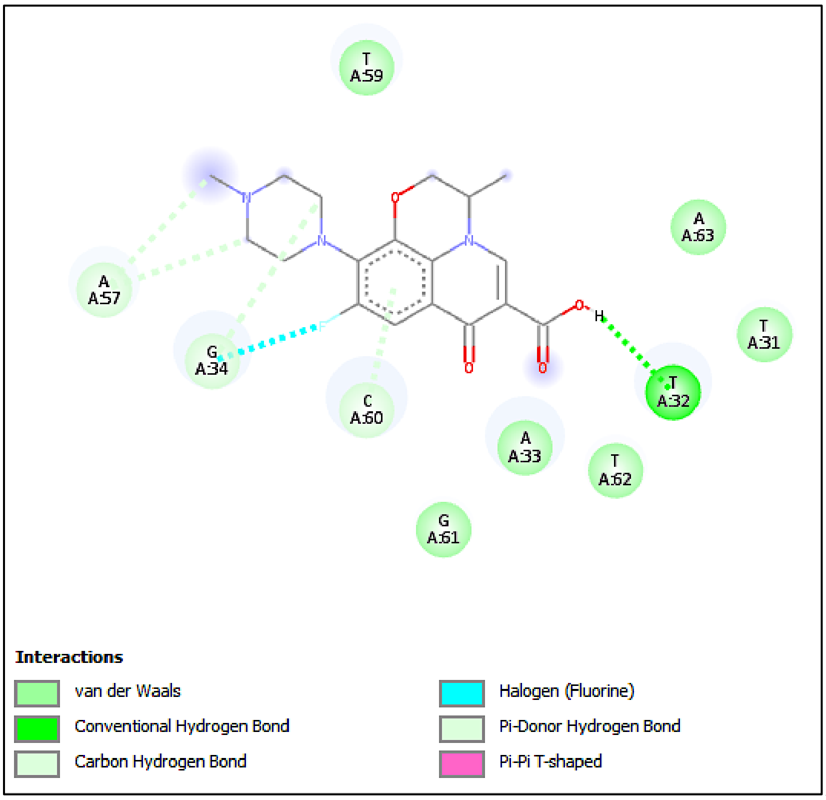

Figure S1 Types of interactions between ofloxacin and aptamer upon recognition (model 1), as shown by DS visualizer (First letter indicates the nucleotide type, A is referred to Aptamer, and the number states the nucleotide's position in the aptamer sequence).

2. Apta-assay optimization

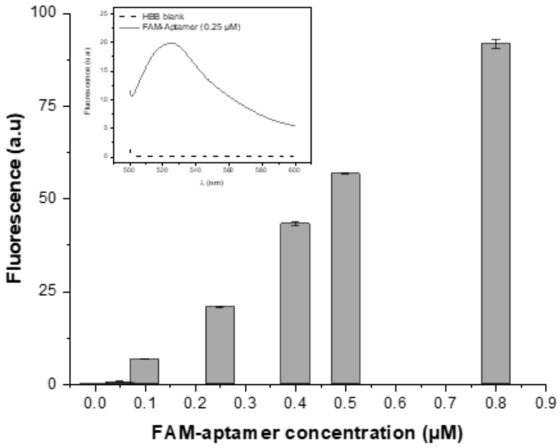

Figure S2 Optimization of initial FAM-Aptamer concentration.
